# Forecast of Healthcare Facilities and Health Workforce Requirements for the Public Sector in Ghana, 2016–2026

**DOI:** 10.15171/ijhpm.2018.64

**Published:** 2018-08-07

**Authors:** James Avoka Asamani, Margaret M. Chebere, Pelham M. Barton, Selassi Amah D’Almeida, Emmanuel Ankrah Odame, Raymond Oppong

**Affiliations:** ^1^Human Resources Division, Ghana Health Service, Accra, Ghana.; ^2^Health Economics Unit, University of Birmingham, Birmingham, UK.; ^3^World Health Organization (WHO), Accra, Ghana.; ^4^Ministry of Health, Accra, Ghana.

**Keywords:** Health Workforce Forecasting, Health Modelling, Health Resources for Health, Healthcare Facilities, Universal Health Coverage

## Abstract

**Background:** Ghana is implementing activities towards universal health coverage (UHC) as well as the attainment of the health-related Sustainable Development Goals (SDGs) by the health sector by the year 2030. Aside lack of empirical forecast of the required healthcare facilities to achieve these mandates, health workforce deficits are also a major threat. We therefore modelled the needed healthcare facilities in Ghana and translated it into year-by-year staffing requirements based on established staffing standards.

**Methods:** Two levels of modelling were used. First, a predictive model based on Markov processes was used to estimate the future healthcare facilities needed in Ghana. Second, the projected healthcare facilities were translated into aggregate staffing requirements using staffing standards developed by Ghana’s Ministry of Health (MoH).

**Results:** The forecast shows a need to expand the number/capacity of healthcare facilities in order to attain UHC. All things being equal, the requisite healthcare infrastructure for UHC would be attainable from 2023. The forecast also shows wide variations in staffing-need-availability rate, ranging from 15% to 94% (average being 68%) across the various staff types. Thus, there are serious shortages of staff which are worse amongst specialists.

**Conclusion:** Ghana needs to expand and/or increase the number of healthcare facilities to facilitate the attainment of UHC. Also, only about 68% of the health workforce (HWF) requirements are employed and available for service delivery, leaving serious shortages of the essential health professionals. Immediate recruitment of unemployed but qualified health workers is therefore imperative. Also, addressing health worker productivity, equitable distribution of existing workers, and attrition may be the immediate steps to take whilst a long-term commitment to comprehensively address HWF challenges, including recruitments, expansion and streamlining of HWF training, is pursued.

## Background

Healthcare delivery across the world, especially in low- and middle-income countries is aimed at universal health coverage (UHC) and most recently, the Sustainable Development Goals (SDGs) by the year 2030.^1^ Central to the achievement of both aspirations is the critical role of a health workforce (HWF), also termed as human resources for health (HRH).^2^ Consequently, human resources for health planning (HRHP) has been identified as an important process towards the attainment of SDG 3 particularly target 3.c that seeks to substantially increase the recruitment, development, training and retention of the health workforce (HWF). In 2015, the World Health Organization (WHO) in consultation with various stakeholders developed a Global Strategy on HWF - Workforce 2030 to respond to HWF challenges across health systems.^3^ It also reinforced the view that the quality and cost of healthcare delivery largely depends on the availability and equitable distribution of health personnel.^4^

The process of HRHP involves determining and putting in place strategies to obtaining the required number of HWF with the right skills and competency; and their appropriate deployment to deliver timely and affordable services that address population health needs.^5,6^ HWF forecasting is one of the initial elements of a broader HRHP.^7^ It encompasses taking stock of available HWF, and estimating current and future HWF needed and comparing with the expected supply. This helps to establish demand and supply gaps (labour market gaps) or current need-availability gaps.^2,7^

There are two sides to HWF forecasting which are HWF supply and HWF demand forecasting.^8^ HWF supply side forecasting involves determining the inflow and outflow of health workers from the current workforce. The inflow depends on the training capacity and immigration, whilst outflow/attrition depends on retirements, deaths, resignations, emigration and dismissals.^2^ On the other hand, HWF demand side forecasting, which is the thrust of this paper, involves determining the current and future HWF requirements.

Commonly used approaches to HWF demand side forecasting include population health needs or epidemiological approach; service demand or utilisation approach; service targets approach; staff-to-population ratios approach; econometrics approach; and health service development analysis (HeSDA)/staffing standards (also known as facility-based) approach.^2,4,6,9,10^ A comprehensive description of these approaches, including their advantages and disadvantages abounds in the literature.^2,7,11^ These models tend to differ in their level of transparency, data requirements and outputs. Therefore, the choice of a particular approach is often informed by the capacity of the analysts, availability of data and the nature of the healthcare system^4^ as there appear to be no method that is superior in all circumstances.

Fakhri and colleagues^12^ compared three methods for HWF forecasting that include the population-needs method, the service-utilisation approach and the service-targets method. The authors reported that the population-needs method yielded a staffing requirement that was 44%-57% higher compared to the service utilisation approach and the service-targets method yielded 10%-21% higher staffing requirements than the service utilisation method.

Also, a Thai study^10^ that compared the projections of future demand for nurses using the staff-to-population ratio, population health needs, and HeSDA showed that although some variation in the estimates was generally seen amongst the three methods, the difference decreased with increasing time horizon (for example, reducing from 40 000 in 2005 to just 10 000 by 2015) and the estimates converged at the end of the forecast period. This partly suggests that some of the HRH forecasting approaches tend to converge or complement each other when used for long term planning.

HRHP in many countries is often done on ad hoc basis with poor data and of varying quality and horizon of planning.^2^ The resultant effects are defective HWF policies that lead to periodic HWF excesses and shortages. An excess in HRH results in economic inefficiencies and supplier-induced demand^2,13^ while HWF shortage is associated with avoidable medical errors, poor and inequitable healthcare delivery.^2^

As espoused in Ghana’s policies, strategic documents and operational plans, the main goal of the health sector is to build a robust health system towards the attainment of UHC.^14-16^ Over the years, the government of Ghana has invested in healthcare infrastructure and health insurance coverage in a bid to improve access to, and bridge inequalities in, healthcare delivery. However, there has not been an empirical forecast of the number and types of health facilities required to attain UHC so as to guide infrastructural investment and distribution of HWF.

Ghana has made some progress in training and retaining HWF in recent years which has culminated in almost doubling the HWF density from 1.07 in 2005 to 2.14 in 2015.^17,18^ Some reports and published literature, however, show that the available HWF do not meet international benchmarks.^19-21^ For instance, the work of Scheffler and colleagues^21^ showed serious deficits in the number of physicians, nurses and midwives in Ghana by 2015, a concern corroborated by operational surveys and annual holistic assessment reports of the Ministry of Health (MoH), Ghana.^22-25^ As part of efforts to address the aforesaid challenges, the MoH developed staffing standards (also known as staffing norms) for healthcare facilities in the country^26^ based on a meta-analysis of individual health facilities’ results of a country-wide staffing study using an evidence-based tool recommended by the WHO, known as Workload Indicators of Staffing Needs (WISN).^27^ Even though the staffing norms have been widely received by stakeholders and operationalised for deployment of newly recruited staff since 2014, the MoH’s holistic assessment report of the health sector programme of work for 2013 recommended “…an analysis of the workforce requirements based on the newly developed staffing norm, and budget forecast ….”^24^ Such a forecast on a year-by-year basis would enhance effective annual planning and budgeting as well as promote responsive HWF policies. This paper focused on forecasting the healthcare facilities and HWF requirements for the public sector in Ghana. In so doing we sought to address the following questions:

1. How many health facilities are needed in the public health sector of Ghana to facilitate the attainment of UHC?
2. Based on the projected health facilities, what would be the HWF requirements of the public health sector of Ghana on a year-by-year basis over the next decade?
3. What is the gap between current staffing levels and the required staffing needs of the public health sector of Ghana?

## Methods

### Introduction to Ghana’s Healthcare Delivery Model

**Figure 1:**
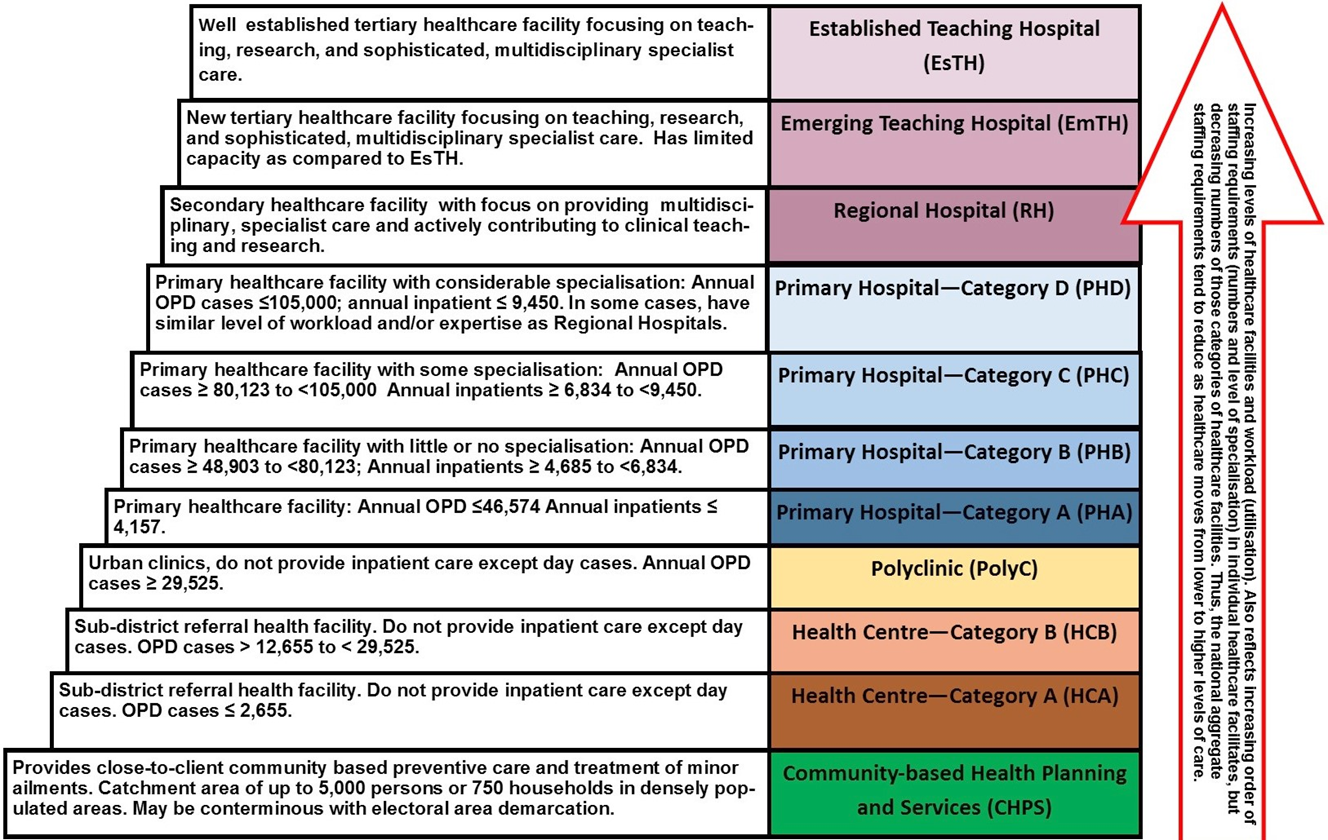
Categorisation of Public Healthcare Facilities in Ghana for Health Workforce (HWF) Allocation. Adapted from Staffing Norms MoH, 2015.

Ghana operates a multi-level public healthcare delivery system (Figure 1). The top tier healthcare delivery institutions are made of autonomous teaching hospitals (THs), which are national referral hospitals with a mandate for managing complex health problems, research and staff training. Each TH is linked with a university to enhance its functions.

There are also regional hospitals (RHs), which provide a secondary level of specialised healthcare and serve as referral centres for each of the ten political regions. The catchment population of RHs is about 1.2 million people.

At the district level, district (primary) hospitals (DHs) serve as referral centres and provide basic and emergency healthcare to populations of 100 000-200 000.^17^ Each district is further divided into health sub-districts which are served by health centres (HCs) that provide basic curative and preventive services covering up to 20 000 population. In urban areas, their capacity is often enhanced and they are then known as polyclinics to serve populations larger than 20 000.

At the bottom of the hierarchy of health service delivery are the community-based health planning and services (CHPS) compounds/zones which is the main strategy for delivering basic primary healthcare at the community level.^14^ These are mandated to provide mainly preventive services and treatment of minor ailments with over-the-counter medications to populations up to 5000 or 750 households. In addition, there are public specialized hospitals, quasi government hospitals and private for profit hospitals and clinics.

### Overview of Modelling Approach

The HeSDA and staffing norms, also known as facility-based approach was selected for Ghana’s context. The HeSDA approach was deemed appropriate for this forecast because it has been shown to be relatively simple but methodologically fit-for-purpose in developing countries.^7,10^ It also accounts for the country’s existing infrastructural capacity whilst allowing for incorporation of plans for future infrastructural and technological expansion, which are key variables in pragmatic HWF forecasts. In Ghana, the MoH has been desirous of forecasting the HWF requirements “within the framework of [the MoH’s] agreed staffing norms.”^15^ In the literature, Kolehmainen-Aitken^7^ have emphasised the importance of this approach, stating, “estimating HWF requirements is [should be] based on the acceptance of norms or standards… [even though] no optimally ‘correct’ standards exist” (p. 16).

This approach estimates future HWF requirements “from projected [number of] healthcare facilities and staffing norms [standards].”^10^ It allows analysts to model future developments of healthcare facilities (in terms of numbers and categories of facilities) based on which predetermined staffing standards are applied to generate aggregate HWF requirements at regional or national levels.

Two levels of modelling were employed to arrive at aggregate HWF requirements. First, Markov state-transition processes were used to forecast the number and categories of future healthcare facilities. MoH staffing norms were then used to translate the number and categories of future health facilities into HWF requirements. These two levels of modelling are described subsequently.

### 1. Modelling the Number of Healthcare Facilities (Health Services Development)

#### Model Structure and Assumptions

**Figure 2:**
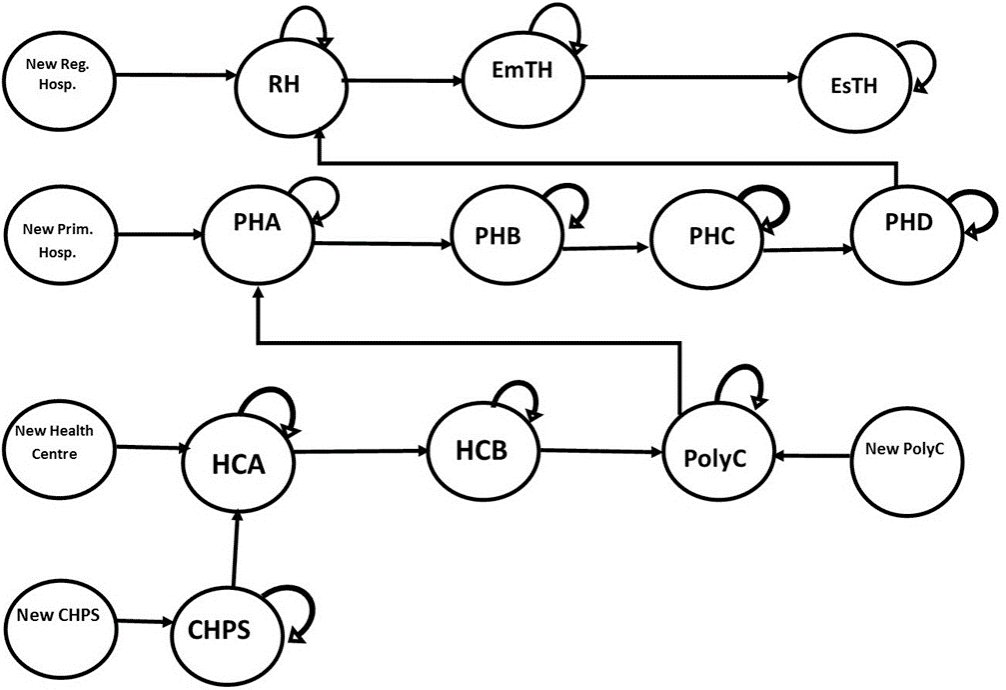
Structure of the Predictive Model for Healthcare Facilities in Ghana.
Source: Authors’ construction.

Abbreviations: CHPS, community-based health planning and services; HCA, health centre in workload category A; HCB, health centre in workload category B; PolyC, polyclinic; PHA, primary hospital in workload category A; PHB, primary hospital in workload category B; PHC, primary hospital in workload category C; PHD, primary hospital in workload category D; RH, regional hospital; EmTH, emerging teaching hospital; EsTH, established teaching hospital.

For the purpose of HWF allocation, publicly funded healthcare facilities are categorised based on their outputs and workload levels (Figure 2). As population and/or workload increases, lower level healthcare facilities would have to be upgraded to higher level categories to enhance their capacity and receive higher HWF allocation.^15,26^

This transition from one category of healthcare facilities to another is analogous to patients moving from one health state to another in typical Markov models as used in health economic evaluations.^28,29^ Therefore, Markov process was deemed appropriate for predicting the future development of health facilities. However, since annual planning cycle is a discrete-time process, half cycle correction was not applicable.

The model deems each category of healthcare facilities as a ‘state’ in which there is a probability of transitioning to another ‘state’ or category of healthcare facility in the future depending on changes in their output and workload levels. Even though transitions from higher categories of healthcare facilities to lower ones are theoretically possible, we conservatively assumed that healthcare facilities can only transition from lower categories to the next higher ones or remain in the same category. This assumption is informed by experiences in the Ghanaian health sector that HWF is not usually withdrawn from healthcare facilities on the basis of a decreased utilisation in a particular year. It is further assumed that existing public healthcare facilities would not be closed down due to high unmet health needs.^17^

The model also takes into account government’s ongoing projects or future plans of establishing new healthcare facilities or expanding existing ones. Figure 2 illustrates the predictive model structure.

#### Time Horizon and Cycle Length

The model was set up to run a cycle of one year for a 10-year forecast horizon. The 1-year cycle was informed by the fact that healthcare facilities’ workload data is usually analysed on an annual basis to inform staff recruitment and distribution planning for the ensuing year.^15^ A 10-year time horizon was chosen to coincide with the lifespan of key strategic policies and plans.^30^ It has also been suggested in the literature that HWF forecasts tend to lose their value beyond ten years due to the rapidly changing dynamics of the healthcare industry.^4^

#### Transition Probabilities

Decision analytic models (DAMs) are driven by transition probabilities, which are defined in this context as the likelihood that a healthcare facility would in the future move from one category or ‘state’ to the other.^29^ The transition probabilities was derived from routine data of healthcare facilities’ utilisation (2011–2015) obtained from the District Health Information Management System database (DHIMS-2) of the MoH.^31^ On a year-by-year basis from 2011 to 2015, each healthcare facility was assigned its appropriate workload category as set out in the MoH staffing norm (see Figure 1 for criteria). The number of transitions from one category of healthcare facility to another were analysed to derive transition probabilities with the aid of Microsoft® Excel (2016 version) and appropriate statistical formulae.^29,32,33^ The derived transition probabilities are shown in Table 1.

**Table 1:** Model Input Parameters

**Parameter**		**Base Value**	**Standard Error**	**Source**
**Transition probabilities**				
	From CHPS to Health Centre – A	0.010	0.001	DHIMS-2
	From Health Centre – A to Health Centre – B	0.033	0.002	DHIMS-2
	From Health Centre – B to Polyclinic	0.028	0.002	DHIMS-2
	From Polyclinic to Primary Hospital – A	0.042	0.002	DHIMS-2
	From Primary Hospital – A to Primary Hospital – B	0.167	0.005	DHIMS-2
	From Primary Hospital – B to Primary Hospital – C	0.129	0.004	DHIMS-2
	From Primary Hospital – C to Primary Hospital-D	0.084	0.003	DHIMS-2
	From Primary Hospital – D to Regional Hospital	0.021	0.002	DHIMS-2
	From Regional Hospital to EmTH	0.091	0.003	DHIMS-2
	From EmTH to EsTH	0.001	0.000	DHIMS-2

Abbreviations: CBPS, community-based health planning and services; EmTH, emerging teaching hospital; EsTH, established teaching hospital; DHIMS-2, District Health Information Management System database.

#### Existing Number of Healthcare Facilities and Transitions From One Category to Another

The number of existing healthcare facilities in each of the ten regions of Ghana as of March, 2016 was taken from the Health Sector Holistic Assessment Report^23^ and the DHIMS-2 database.^31^ Ongoing and planned projects of establishing new healthcare facilities or expansion of existing ones were taken from various government sources.^15,34,35^ Data on the number of demarcated CHPS zones in various regions was based on extrapolation from the CHPS policy (1500 population or 750 households per CHPS zone)^14^ using population estimates (see equation I).

Based on the possible movements of health facilities from one workload category to another as depicted in the model structure (Figure 2) and the associated transition probabilities (Table 1), the following set of formulae (equations II–XI) was used to compute the number of health facilities in each year.

*CHPS*_i,j_ *= Population*_i,j_ */1,500* **Eq. (I)**

HCA_i,j_ = (HCA_i, j-1_ – (HCA_i, j-1_ * Transition Probability _HCA to HCB_)) + (CHPS_i, j-1_ * Transition Probability _CHPS to HCA_) + Newly constructed HC **Eq. (II)**

HCB_i,j_ = (HCB_i, j-1_ – (HCB_i, j-1_ * Transition Probability _HCB to PolyC_)) + (HCA_i, j-1_ * Transition Probability _HCA to HCB_) **Eq. (III)**

PolyC_i,j_ = (PolyC_i, j-1_ – (PolyC_i, j-1_ * Transition Probability _PolyC to PHA_)) + (HCB_i, j-1_ * Transition Probability _HCB to PolyC_) + Newly constructed PolyC **Eq. (III)**

PHA_i,j_ = (PHA_i, j-1_ – (PHA_i, j-1_ * Transition Probability _PHA to PHB_)) + (PolyC_i, j-1_ * Transition Probability _PolyC to PHA_) + Newly constructed PHA **Eq. (V)**

PHB_i,j_ = (PHB_i, j-1_ – (PHB_i, j-1_ * Transition Probability _PHB to PHC_)) + (PHA_i, j-1_ * Transition Probability _PHA to PHB_) **Eq. (VI)**

PHC_i,j_ = (PHC_i, j-1_ – (PHC_i, j-1_ * Transition Probability _PHC to PHD_)) + (PHA_i, j-1_ * Transition Probability _PHB to PHC_) **Eq. (VII)**

PHD_i,j_ = (PHD_i, j-1_ – (PHD_i, j-1_ * Transition Probability _PHD to RH_)) + (PHC_i, j-1_ * Transition Probability _PHC to PHD_) **Eq. (VIII)**

RH_i,j_ = (RH_i, j-1_ – (RH_i, j-1_ * Transition Probability _RH to EmTH_)) + (PHD_i, j-1_ * Transition Probability _PHD to RH_) + Newly constructed RH **Eq. (IX)**

EmTH_i,j_ = (EmTH_i, j-1_ – (EmTH_i, j-1_ * Transition Probability _EmTH to EsTH_)) + (RH_i, j-1_ * Transition Probability _RH to EmTH_) + Newly constructed EmTH **Eq. (X)**

EsTH_i,j_ = EsTH_i, j-1_ + (EmTH_i, j-1_ * Transition Probability _EmTH to EsTH_) **Eq. (XI)**

Where:

CHPS_i,j_ represents the number of CHPS in administrative region *i* at year *j*.

J-1 represents the previous year.

Transition Probability_HCA to HCB_ represents the probability of Health Center A transitioning to Health Center B in a given year.

Similar notations apply to all the categories of healthcare facilities.

### 2. Translating the Number of Healthcare Facilities Into HWF Requirements

#### Computing the Health Workforce Requirements

To get the required HWF, the projected number of various categories of healthcare facilities in a particular year was multiplied by the appropriate staffing norm. This was then adjusted for workload changes. The HWF requirement was calculated using the following formula:

HWFk_ij_ = ∑[(CHPS_ij_*SSk_CHPS_) + (HCA_ij_*SSk_HCA_) + (HCB_ij_*SSk_CHB_) + (PolyC_ij_*SSk_PolyC_) + (PHA_ij_*SSk_PHA_) + (PHB_ij_*SSk_PHB_) + (PHC_ij_*SSk_PHC_) + (PHD_ij_*SSk_PHD_) + (RH_ij_*SSk_RH_) + (EmTH_ij_*SSk_EmTH_) + (EsTH_ij_*SSk_EsTH_)] Eq. (XII)

Where:

HWFk_ij_ represents the base HWF requirement for a particular type of staff *k* in administrative region *i* at year *j*.

*SSk*_CHPS_ represents the stipulated staffing standard for staff type *k* at the CHPS level (similar notations apply to the other categories of healthcare facilities example, HCA, HCB etc.)

CHPS_ij_ represents total number of CHPS in region *i* at year *j*.

The adjusted HWF requirement for region *i* at year *j* (denoted *HWF*_ijadj_*)* take the form**:**

*HWF*_ijadj_ *= ∑ [HWF*_ij_ *+ (HWF*_ij_ *wca)] **Eq. (XIII)**

As stipulated by the MoH staffing norms, the base HWF requirement was adjusted for workload changes that require adjustments in staffing level (denoted *wca*) but not significant to lead to a healthcare facility moving from one workload category to another.

The national HWF requirements then become a summation of the HWF requirements from all the ten political/administrative regions of Ghana.

##### The Ghana Staffing Standards/Norms

The staffing standards applied in the formulae above was taken from the MoH staffing norms for the health sector of Ghana, volume 1.^26^ Staffing standards define expected workload levels at various categories of health facilities, and the number of each cadre of health staff required in those healthcare facilities to deliver healthcare. The number of staff required in various health facilities as per the staffing norms is publicly available.^27^

The staffing norms^26^ also provide that when the workload in a health facility changes beyond 5% but the change is not sufficient to cause a transition of a health facility from one category of HWF requirement to another, the following guide should be used for adjustments.

- 15% or more change in workload would lead to about 23.5% adjustments in staff requirement [however, workload changes beyond 15% require a thorough assessment of the status of the health facility].
- Above 10% but less than 15% change in workload would lead to about 14.3% adjustment in staff requirement.
- 5% up to 10% change in workload would lead to about 6.3% adjustment in staff requirement.
- Less than 5% change in workload does not merit adjustment in staff requirement.

The aforesaid policy guide was incorporated in the model.

##### Determining Existing Staff-Availability Gaps

To determine the existing level of HWF needs-availability gaps, a ratio of the current staffing levels to the projected requirement was made (referred to as staff-availability ratio, SAR). Two forms of HWF gap analysis can be distinguished. First, supply-side and demand-side HWF forecasts can be compared to establish the existence or otherwise of labour market equilibrium or supply-and-demand gap.^36^ This is often useful for planning training and development programmes. The second, known as the HWF needs-availability gap where existing staffing levels (those employed) are compared with projected needs is applied in this study. This type of gap analysis is useful for recruitment and deployment planning as well as wage bill management.^7^

##### Sensitivity Analysis

Uncertainty around the point estimates of HWF requirements arising from parameter uncertainty, particularly the transition probabilities were explored by simultaneously varying all the transition probabilities in the predictive model using both their lower and upper 95% confidence limits. Savelli and Joslyn^37^ point out that “uncertainty can be expressed as a predictive interval, providing the upper and lower boundaries of a range within which the observed value is expected.”

## Results

### Modelled Transitions of Health Facilities From One Category of Workload to the Other

Using equations II–XI, we modelled the expected transition of health facilities from one category the other (Table 2). The forecast shows marginal annual changes in the number of health facilities making transitions from one workload category to the other. Notably, only transitions from primary hospitals in workload categories A (PHA) to category B (PHB) is predicted to take place at a declining rate from 16 in 2016 to about 6 in 2026. In aggregate, over the next decade, it is expected that about 637 CHPS will likely transition to health centre within workload category A (HCA) while some 369 HCA will also transition to HCB but only 89 HCBs are expected to move to become Polyclinics (PolyC) within the next decade. Also, a near equilibrium is expected between the number of health facilities transitioning from PHD to RH and those from RH to EmTH.

**Table 2:** Modelled Transitions From One Category of Health Facility to Another

**Year**	**CHPS to HCA**	**HCA to HCB**	**HCB to PolyC**	**PolyC to PHA**	**PHA to PHB**	**PHB to PHC**	**PHC to PHD**	**PHD to RH**	**RH to EmTH**	**EmTH to EsTH**
2016	45.6	29.5	4.5	1.7	16.0	6.3	2.4	0.3	0.6	-
2017	48.8	30.4	5.2	2.0	14.4	7.6	2.8	0.3	0.6	-
2018	52.1	31.1	5.9	2.3	13.0	8.4	3.2	0.4	0.7	-
2019	55.4	31.8	6.6	2.7	11.8	9.0	3.6	0.4	0.6	
2020	58.7	32.6	7.3	2.9	10.4	9.4	4.1	0.5	0.6	-
2021	59.8	33.4	8.0	3.1	9.2	9.5	4.5	0.6	0.6	-
2022	60.9	34.3	8.8	3.3	8.2	9.5	4.9	0.7	0.6	-
2023	62.0	35.2	9.5	3.5	7.3	9.3	5.3	0.8	0.6	-
2024	63.2	36.0	10.2	3.8	6.7	9.1	5.7	0.9	0.6	-
2025	64.4	36.9	10.9	4.0	6.2	8.8	5.9	1.0	0.7	-
2026	65.6	37.8	11.7	4.3	5.9	8.4	6.2	1.1	0.7	-
Total Number of transitions	637	369	89	34	109	95	49	7	7	

Abbreviations: CHPS, community-based health planning and services; HCA, health centre in workload category A; HCB, health centre in workload category B; PolyC, polyclinic; PHA, primary hospital in workload category A; PHB, primary hospital in workload category B; PHC, primary hospital in workload category C; PHD, primary hospital in workload category D; RH, regional hospital; EmTH, emerging teaching hospital; EsTH, established teaching hospital.

### Healthcare Facilities (Service Development) Forecast

Overall, the forecast shows the need for an increase of about 45% in the aggregate number of healthcare facilities from 5749 at baseline to 8326 by 2026 (Table 3). Such steady increase in the number of healthcare facilities is predicted across all the categories of healthcare facilities except RHs and primary hospitals in workload categories A and B (PHA and PHB). In particular, the predicted number of PHAs tends to decrease steadily from 96 at baseline (2016) to 35 by 2026 because their workload is expected to increase whereby many of them would expand and move into the next higher category (PHB). Consequently, the number of primary hospitals in workload category B (PHB) is predicted to steadily increase from a baseline total of 49 and reach a peak of 74 by the fifth and sixth year (2021/2022), and thereafter, decline marginally (7%) to 65 by the year 2026 as they expand and move into category C (PHC). However, the total number of primary hospitals is expected to increase by 20% from 188 in 2016 to 225 by the year 2026. This would potentially enable the attainment of a primary hospital in each district as may be necessary for UHC.

**Table 3:** Healthcare Facilities (Categories and Number), 2017–2026

**Health Facility Type/Year**	**Baseline (2016)**	**2017**	**2018**	**2019**	**2020**	**2021**	**2022**	**2023**	**2024**	**2025**	**2026**
CHPS	4449	4768	5088	5409	5731	5835	5943	6054	6169	6287	6409
HCA	903	930	953	975	998	1024	1051	1077	1104	1131	1159
HCB	158	183	208	233	259	284	309	335	360	386	412
PolyC	40	48	56	66	69	74	79	84	90	97	103
PHA	96	87	78	71	63	55	49	44	40	37	35
PHB	49	59	66	70	73	74	74	72	70	68	65
PHC	29	33	38	43	48	54	59	63	67	71	73
PHD	14	16	19	21	25	28	32	36	41	46	51
RH	7	7	7	7	7	7	7	7	7	7	7
EmTH	2	3	3	4	4	4	5	5	6	7	7
EsTH	2	2	2	2	3	3	3	3	3	3	3
Total Number of Primary (District) Hospitals	188	194	200	205	208	211	213	216	219	222	225
**Total Number of Health Facilities**	5749	6135	6518	6901	7279	7442	7610	7782	7958	8140	8326

Abbreviations: CHPS, community-based health planning and services; HCA, health centre in workload category A; HCB, health centre in workload category B; PolyC, polyclinic; PHA, primary hospital in workload category A; PHB, primary hospital in workload category B; PHC, primary hospital in workload category C; PHD, primary hospital in workload category D; RH, regional hospital; EmTH, emerging teaching hospital; EsTH, established teaching hospital.

On the other hand, the forecasts show that RHs are expected to remain at a total of 7 throughout the forecast period mainly because the number of hospitals moving into that category tends to be similar to those expected to be developed and re-designated as emerging teaching hospitals (EmTHs). Consequently, the number of EmTHs is predicted to increase from 2 at baseline to 7 by 2026 (about a 250% increase) whereas established teaching hospitals (EsTHs) would increase from 2 to 3 during the same period. Thus, the forecast shows a need to have a total of 10 teaching hospitals (EmTH and EsTH) by 2026, potentially one for each of the 10 political/administrative regions.

### Forecast of Aggregate HWF Requirements and Existing Staff-Availability Gaps

**Figure 3:**
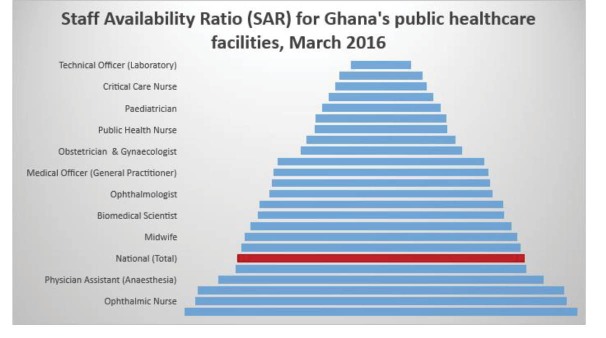
Staff-Availability Ratio (SAR) in Ascending Order of Existing Staffing Deficit (March 2016).

Generally, the results show a steady increase in HWF requirements across staff types are required to enable effective healthcare delivery in the public healthcare facilities (see Table 4 for the aggregate HWF requirements). Also, the forecast of the HWF requirements at the sub-national level was conducted for all the ten regions of Ghana (see Supplementary file 1 – Tables S1-S10). We also compared the existing number of staff employed by the MoH (current staffing levels) as a proportion of the projected requirements. The resulting SARs are presented in Figure 3. In general, the average SAR (as of March 2016) was 68% but ranges widely from 15% to 94% across various staff types. Nine out of the 23 (39%) staff types considered in the forecast have SAR between 50% and 69% whilst only four (17%), including community health nurse, enrolled nurse, ophthalmic nurse, and physician assistant-anaesthesia are 70% or more. Conversely, the SAR of majority (10 out of 23) or 44% of the staff types are less than 50% and are considered to be in severe shortage.

**Table 4:** Aggregate HWF Requirements for the Public-Sector Healthcare Facilities in Ghana, 2016–2026

**NO.**	**Staff Type**	**Aggregate HWF Requirement for the Year**										
		**2016**	**2017**	**2018**	**2019**	**2020**	**2021**	**2022**	**2023**	**2024**	**2025**	**2026**
1	Biomedical scientist	1392	1548	1719	1894	2146	2313	2488	2670	2861	2933	3006
2	Community health nurse	17 559	19 441	21 380	23 432	25 506	27 062	28 678	30 355	32 095	32 911	33 728
3	Critical care nurse	1375	1537	1717	1901	2215	2407	2607	2817	3038	3115	3192
4	Dental surgeon	237	254	273	289	305	321	339	357	377	386	396
5	Emergency nurse	1145	1279	1435	1590	1804	1967	2139	2321	2511	2575	2639
6	Enrolled nurse	17 315	19 258	21 207	23 363	25 418	27 221	29 068	30 961	32 902	33 739	34 576
7	Family medicine physician	251	268	287	303	323	337	352	367	384	394	404
8	General surgeon	280	301	326	348	377	399	422	447	474	486	498
9	Medical officer (general practitioner)	3094	3434	3719	4194	4610	5004	5416	5846	6294	6454	6614
10	Mental health nurse	2034	2236	2347	2659	2879	3098	3325	3560	3803	3900	3997
11	Midwife	8816	9918	10 886	12 247	13 554	14 718	15 906	17 121	18 365	18 832	19 299
12	Obstetrician & gynaecologist	490	547	611	673	750	814	880	948	1018	1044	1070
13	Ophthalmic nurse	434	487	544	601	659	708	758	810	864	886	908
14	Ophthalmologist	85	95	111	125	147	164	183	202	222	228	233
15	Paediatrician	444	496	553	609	675	731	788	846	906	929	952
16	Pharmacist	982	1089	1213	1334	1502	1620	1743	1871	2004	2055	2106
17	Pharmacy technician	3640	4045	4368	4903	5360	5794	6244	6712	7197	7380	7563
18	Physician assistant (anaesthesia)	759	836	929	1014	1108	1197	1290	1387	1489	1527	1565
19	Physician assistant (medical)	2282	2539	2709	3087	3353	3633	3926	4233	4554	4670	4786
20	Public health nurse	839	942	1055	1165	1271	1376	1483	1594	1708	1751	1795
21	Radiographer/x-ray technician	719	798	885	971	1062	1141	1225	1311	1401	1437	1473
22	Registered general nurse	21 971	24 477	26 902	29 888	33 428	36 119	38 888	41 744	44 692	45 829	46 966
23	Technical officer (laboratory)	3501	3924	4369	4832	5235	5643	6068	6510	6968	7145	7322
	**Total**	89 644	99 749	109545	121 420	133 686	143 786	154 217	164 991	176 125	180 606	185 087

Abbreviation: HWF, health workforce.

### Sensitivity Analysis

As described in the method section, uncertainties around the base estimates in HWF requirements were characterised in the form of predictive intervals which provides a plausible range within which we are 95% confident the true value would lie (see Table 5). The results show narrow predictive intervals, suggesting only minimal-to-moderate level of uncertainty within the forecast.

**Table 5:** 95% Prediction Interval for The Aggregate HRH Requirements

**No.**	**Type of Staff**	**95% Predictive Interval by Year**																			
		**2017**		**2018**		**2019**		**2020**		**2021**		**2022**		**2023**		**2024**		**2025**		**2026**	
		**Lower**	**Upper**	**Lower**	**Upper**	**Lower**	**Upper**	**Lower**	**Upper**	**Lower**	**Upper**	**Lower**	**Upper**	**Lower**	**Upper**	**Lower**	**Upper**	**Lower**	**Upper**	**Lower**	**Upper**
1	Biomedical scientist	1542	1555	1706	1733	1872	1917	2023	2088	2177	2264	2337	2449	2503	2643	2676	2847	2744	2920	2812	2992
2	Community health nurse	19 397	19 485	21 295	21 466	23 285	23 578	25 286	25 698	26 777	27 320	28 323	29 005	29 925	30 757	31 584	32 577	32 388	33 406	33 192	34 235
3	Critical care nurse	1529	1544	1701	1732	1876	1927	2046	2119	2222	2320	2405	2531	2596	2753	2796	2987	2867	3063	2938	3139
4	Dental surgeon	253	254	272	273	288	291	303	308	318	325	334	343	351	363	370	384	379	394	388	404
5	Emergency nurse	1273	1284	1422	1447	1569	1611	1714	1774	1864	1946	2023	2128	2190	2321	2365	2525	2425	2589	2485	2653
6	Enrolled nurse	19 192	19 324	21 075	21 338	23 150	23 576	25 123	25 712	26 839	27 603	28 594	29 543	30 390	31 534	32 228	33 578	33 048	34 432	33 868	35 286
7	Family medicine physician	267	268	287	288	302	305	316	320	329	335	343	351	357	367	372	385	382	395	391	405
8	General surgeon	300	302	325	327	346	350	366	373	386	396	408	421	430	448	455	476	466	488	478	501
9	Medical officer (general practitioner)	3413	3456	3675	3764	4120	4267	4465	4673	4824	5100	5199	5547	5589	6015	5994	6503	6147	6668	6299	6834
10	Mental health nurse	2219	2253	2312	2382	2601	2717	2788	2951	2980	3195	3179	3449	3385	3713	3597	3987	3688	4089	3780	4190
11	Midwife	9854	9982	10 756	11 016	12 040	12 454	13 099	13 673	14 172	14 918	15 264	16 193	16 378	17 501	17 513	18 844	17 959	19 323	18 405	19 803
12	Obstetrician & gynaecologist	544	549	607	616	665	680	724	746	784	812	846	882	910	954	976	1028	1001	1054	1026	1081
13	Ophthalmic nurse	485	489	540	549	594	607	640	659	685	710	732	764	779	820	829	879	850	902	871	924
14	Ophthalmologist	95	96	109	112	123	128	138	145	155	163	172	183	190	203	209	224	214	230	220	236
15	Paediatrician	494	498	549	557	602	615	655	673	708	731	762	791	817	852	873	914	895	938	917	961
16	Pharmacist	1085	1094	1204	1223	1318	1349	1426	1470	1535	1594	1648	1724	1765	1859	1887	2001	1935	2052	1983	2103
17	Pharmacy technician	4018	4072	4312	4423	4812	4994	5194	5452	5586	5926	5992	6420	6411	6934	6844	7469	7018	7659	7192	7849
18	Physician assistant (anaesthesia)	834	839	922	935	1004	1024	1086	1115	1170	1209	1258	1307	1349	1410	1444	1518	1481	1556	1518	1595
19	Physician assistant (medical)	2517	2560	2664	2754	3013	3161	3249	3459	3495	3773	3751	4103	4018	4449	4297	4813	4406	4935	4515	5058
20	Public health nurse	938	945	1047	1063	1153	1178	1251	1287	1350	1397	1451	1511	1555	1628	1662	1749	1704	1794	1746	1838
21	Radiographer/x-ray technician	796	801	879	891	961	981	1035	1064	1110	1148	1187	1236	1267	1329	1351	1426	1385	1462	1419	1499
22	Registered general nurse	24 345	24 609	26 630	27 175	29 450	30 326	31 892	33 123	34 365	35 982	36 901	38 937	39 505	41 997	42 183	45 173	43 256	46 323	44 329	47 472
23	Technical officer (laboratory)	3907	3940	4333	4406	4773	4891	5151	5320	5531	5758	5925	6217	6331	6695	6751	7193	6923	7376	7095	7559
	Total	99 297	100 200	108 621	110 470	119 917	122 926	129 970	134 199	139 362	144 924	149 033	156 033	158 994	167 545	169 255	179 484	173 561	184 050	177 867	188 617

Abbreviation: HRH, human resources for health.

## Discussion

### Number of Healthcare Facilities

The forecast shows a need to expand the number and/or capacity of existing healthcare facilities to cope with the expanding service delivery coverage and population increases if Ghana is to achieve its aim of UHC by 2020.^15^ UHC is defined in terms of all people obtaining a range of promotive, preventive, curative, rehabilitative and palliative services according to their needs, and without suffering financial hardship.^38^ This necessarily requires adequate provision of at least primary-level healthcare facilities including CHPS, HCs and DHs for communities, sub-districts and districts respectively. However, given the trend of health service utilisation and pace of service developments, this forecast shows it may take up to 2023/2024 to achieve at least 216 DHs, potentially one for each of the 216 districts. Thus, under the aforesaid assumptions, the goal of attaining UHC by its true definition may only be feasible by 2023 onwards. In terms of financial protection for the poor, Ghana has made significant strides with a social health insurance coverage of 54% of its population (comprising 66% of the poor).^22,39^ However, inadequate healthcare infrastructure and human resources due to underinvestment in the health sector appear to be the key drawbacks to the attainment of UHC.^39^ The government for instance, spent only 9.7% of its national budget on health^39^ against a target of 15%.^15,40^ Of this, only 5% of the health budget was spent on capital and infrastructural investment whilst about 94% was spent on HWF salaries and emoluments.^41^

In contrast to a World Bank report^17^ suggesting there were more DHs in Ghana than required, this forecast has demonstrated a need for an increase. Using a WHO normative standard of one DH per 150 000-250 000 population, and given Ghana’s estimated population of 29 422 275 by 2017, about 196 DHs would be needed. This is similar to the need for 194 predicted by this model. This not only underpins the validity of this model but also supports the need to equitably expand the healthcare infrastructure.

Indeed, the MoH in its capital investment plan has earmarked several health facilities to be upgraded while new ones of various types are at various stages of construction across the country.^42^ The projections in this paper suggests the need for the expansion of healthcare infrastructure although we acknowledge that these investments must be distributed equitably to ensure that no population groups are left behind in the efforts towards UHC.

### HWF Requirements and Gaps

HWF shortages have been predicted and reported across Africa,^19,21^ resulting in various efforts to address it. Whilst Ghana’s efforts have yielded positive results, it continues to face significant HWF challenges.^17,21^ Compared with Scheffler and colleagues’ estimates of HWF shortages by 2015, the current estimates are lower. Whilst this is attributable to fundamental differences in theoretical and methodological approaches, it is instructive to note that both models point to significant gaps in HWF availability in Ghana. Scheffler and colleagues assumed a normative standard of 0.55 doctors and 1.77 nurses and midwives per 1000 population across countries irrespective of infrastructural capacity and the influence of other local factors. Thus, it tends to yield high HWF estimates but also useful for comparison across countries.

This forecast shows an average SAR of 68%, but the figure varies widely by staff type from 15%-94%. Of 23 staff types considered, only four had a SAR of 70% or more, notably auxiliary nurses (enrolled nurses and community health nurses usually trained for 2 years). This is attributable to the government’s deliberate policy to expand and liberalise the training of these categories of health workers.^17,43,44^ This has, however, created a seeming skill-mix distortion in the nursing workforce whereby in 2015 about 57.4% of the clinical nursing staff were auxiliary (enrolled nurses) as against a desired national standard of 40%.^34^ This forecast shows a ratio of 38% enrolled nurses to 62% professional nurses. A ratio of 30%:70% is however recommended in the nursing literature.^45^

On the other hand, the SAR of most generalist health professionals were estimated to be below 70%, a milestone required to attain and sustain essential service provision. However, beyond the general shortage of the essential health workers (doctors, nurses and midwives), a more serious shortage is observed among the specialised professionals. These include among others, emergency nurses, critical care nurses, paediatricians, obstetricians and gynaecologists, as well as family medicine physicians. Whereas the level of shortfall among these specialists is serious, it does not appear that the gaps can be filled in the short term as their training takes a minimum of 2-3 years, and the training institutions also have limited capacity to increase enrolments. Also, the limited availability of generalist professionals meant that not so many can be allowed to take up specialist training at one time. A similar phenomenon of specialist shortages have been reported in other countries such as Spain, Zambia and the United States among other,^46-49^ suggesting a global challenge.

The current forecast has also indicated a severe shortage of some para-clinical staff such as laboratory technicians (85% shortfall), pharmacy technicians (75% shortfall) and radiographers (69% shortfall). However, these have not been given prominence in both the international and local literature, a situation which suggests why a seeming low level of priority is being given to the training of these professionals whose input to quality healthcare delivery is substantial.

### Policy Implications

This study brings to light a number of policy issues. First, there is a need for the government to generally increase and sustain investments in the health sector in the medium-to-long. Significant part of this investment should focus on equitably establishing more health facilities and expanding some of the existing ones to address the growing population health needs towards UHC. Amidst fiscal constraints, the government needs to show greater commitment to the Abuja target of spending at least 15% of the annual national budget on healthcare.^40^

Similarly, there is the need to increase investment in the development, recruitment and retention of the requisite HWF, and ensure appropriate distribution of this investment, to provide the needed services in healthcare facilities. In this regard, the MoH and its service delivery agencies need to urgently define the HWF national priorities by developing a medium-to-long term recruitment, training and development plan for generalist and specialist health professionals. In so doing, attention should also be paid to the so-called neglected para-clinical professionals in short supply.

In the interim, MoH could consider employing graduate level prepared nurses, privately trained physician assistants, pharmacists and foreign trained medical officers who are paradoxically unemployed amidst the need for their services.

Inefficiencies and low productivity have been reported among Ghanaian health workers.^50,51^ However, up to 20% reduction in shortages could be achieved by marginally increasing productivity or altering the staff skill-mix through task-sharing.^21,52^ Therefore, the MoH could embark on developing staff productivity improvement initiatives across health workers, and also explore viable task-sharing options.

### Strengths and Limitations

This work appears to be one of the first attempts to empirically forecast the HWF need of a country based on the HeSDA approach and made use of service data from all healthcare facilities while incorporating existing health sector plans and policies. It also provided the first forecast of the healthcare facilities needed in Ghana over a 10-year horizon. Whereas the forecast provided is specific to Ghana, the model is adaptable to other settings and has relatively moderate data requirements as compared to other approaches.

However, some limitations are worth noting. First, the forecast is limited in scope as it focused on only publicly funded healthcare facilities (government and faith-based) and selected types of staff. The private and quasi-government healthcare facilities, 57% of which are located in Accra and Kumasi Metropolis,^53^ were not considered in the forecast due to data constraints. Thus, when interpreting or using the forecast, one must be reminded that it does not necessarily represent the comprehensive picture of Ghana’s health sector.

Secondly, this is only the HWF demand forecast that did not include HWF supply analysis. Consequently, the gaps presented are not supply-and-demand gaps (labour market equilibrium) but those of the needed staff currently employed; these concepts have separate significance and have been distinguished in the methods section.

Finally, the DHIMS-2 database from which service data was extracted to derive transition probabilities still has some limitations in data quality (95%) and completeness (99.5%) even though timeliness of the data reporting is reportedly 100%.^34^ Thus, the point estimates must be regarded as ordered rough estimates. Therefore, the predictive intervals which account for these uncertainties should always be taken into consideration when using the forecast as a decision-making aid.

## Conclusion

There is a need to expand and/or increase the number of healthcare facilities to facilitate the attainment of UHC. Given the pace of execution of government healthcare infrastructural projects and trends of healthcare utilisation, it is expected that the requisite healthcare infrastructure for UHC would be attained from 2023 onwards.

Ghana has an average of 68% of its HWF requirements, but there are serious shortages of the essential health professionals that are worse amongst the specialists’ groups. Addressing this situation may require a substantial increase in government’s expenditure on HWF in the short-to-medium term, a demand that may be difficult to meet due to fiscal constraints. Under the circumstances, recruitment of trained but unemployed health professionals, improving HWF productivity, and ensuring equitable distribution of existing HWF may be the immediate steps to take whilst a long-term commitment to comprehensively address HWF challenges, including recruitments, expansion and streamlining of HWF training, is pursued.

## Recommendations for Further Research

In taking this work forward, it would be necessary to conduct a supply-side forecast to establish the health labour market (dis)equilibrium in the Ghanaian context to inform future training and development policies. Since supply-side HWF forecast was not considered here, HWF supply and demand gap analysis appears not feasible under the circumstances. However, on an annual basis, the existing staffing levels could be compared with the projected requirements to establish HWF need-availability gaps to facilitate recruitment, distribution, and redeployment planning.

To further strengthen the validity of the approach used, it would be useful to undertake the forecast with alternative approaches for comparison. This would not only enhance the quality of policy decisions but also enrich academic discourse and fill gaps in the literature.

Finally, the model has shown promise in forecasting HWF needs using data from Ghana but there is the need to adapt it for testing and use with data from other countries.

## Ethical issues

The study did not involve human subjects. The first author is an authorised user of the DHIMS-2 and human resources databases in Ghana but additional permission was obtained from Ghana Health Service (GHS) to use aggregate data for the purpose of this forecast. In addition, care was taken to ensure anonymity such that no identifiable information of individual healthcare facility, patient or staff is reported.

## Competing interests

Authors declare that they have no competing interests.

## Authors’ contributions

JAA, PMB, and RO conceived of the study. JAA undertook literature review, JAA, PMB, RO designed the methodology; JAA undertook the modelling under the guidance and supervision of PMB and RO. JAA, MMC, SAD, and EAO drafted the manuscript which all the authors critically reviewed. All authors approved the manuscript for publication.

## Authors’ affiliations

^1^Human Resources Division, Ghana Health Service, Accra, Ghana. ^2^Health Economics Unit, University of Birmingham, Birmingham, UK. ^3^World Health Organization (WHO), Accra, Ghana. ^4^Ministry of Health, Accra, Ghana.

## Supplementary files

Supplementary file 1 contains Tables S1-S10.

## Key messages

**Implications for policy makers**

- Using agreed staffing standards/norms to estimate aggregate health workforce (HWF) requirements for a country could produce realistic projections to guide HWF investments, recruitment, deployment and retention strategies.
- On average, there is about 32% shortage of the required HWF in Ghana. The shortage is however highest (75%) amongst laboratory workers and lowest (6%) amongst auxiliary nurses. Policy-makers in Ghana should reprioritise the training and recruitment of frontline health workers taking into account the emerging evidence.
- Policy-makers in Ghana should give specialist training the needed priority in terms of funding to address the serious specialists’ shortage.

**Implications for the public**

This paper focused on estimating the number of health facilities with corresponding health workforce (HWF) requirements for evidence-informed planning in Ghana. Such evidence would complement efforts to expand service coverage for all populations. Public advocacy is needed to shape the policy agenda for equitable investment in the production and retention of all required categories of the HWF.
