# Supplementary material for: Forecast of Healthcare Facilities and Health Workforce Requirements for the Public Sector in Ghana, 2016–2026
Source: Int J Health Policy Manag. 2018 Aug 7;7(11):1040–52. doi: 10.15171/ijhpm.2018.64 (PMC6326637; doi:10.15171/ijhpm.2018.64)
Supplement: Supplementary file 1 — contains Tables S1-S10. [file ijhpm-7-1040-s001.pdf]

Supplementary Table 1: Aggregate HWF Forecast for Ashanti Region

STAFF TYPE	2016	2017	2018	2019	2020	2021	2022	2023	2024	2025	2026
Biomedical Scientist	316	342	373	408	437	468	500	533	567	582	596
Community Health Nurse	3206	3506	3840	4149	4485	4776	5079	5395	5723	5869	6014
Critical Care Nurse	335	360	394	431	465	501	538	577	618	633	649
Dental Surgeon	44	48	53	58	61	63	67	70	74	75	77
Emergency Nurse	236	260	295	327	355	386	419	454	490	502	515
Enrolled Nurse	3029	3409	3822	4230	4607	4963	5327	5698	6076	6231	6385
Family Medicine Physician	49	54	59	64	66	69	72	74	77	79	81
General Surgeon	56	61	67	73	77	81	85	89	94	96	99
Medical Officer (General Practitioner)	495	560	639	727	799	875	954	1037	1123	1152	1180
Mental Health Nurse	309	345	370	425	462	501	541	583	627	643	659
Midwife	1524	1738	1956	2220	2444	2670	2900	3134	3371	3457	3543
Obstetrician & Gynaecologist	93	105	122	136	148	161	174	187	201	206	211
Ophthalmic Nurse	83	94	106	117	126	135	144	154	164	168	172
Ophthalmologist	15	17	22	24	28	31	35	39	43	44	45
Paediatrician	86	97	110	123	134	145	157	168	180	185	189
Pharmacist	209	228	256	281	303	325	348	372	397	407	417
Pharmacy Technician	615	690	747	846	921	1000	1081	1166	1253	1285	1317
Physician Assistant (Anaesthesia)	134	152	177	197	213	231	250	269	289	296	303
Physician Assistant (Medical)	332	374	407	468	513	561	612	665	721	739	757
Public Health Nurse	147	169	195	219	239	260	281	303	325	333	342
Radiographer /X-ray Technician	138	155	172	190	205	220	236	252	269	276	282
Registered General Nurse	4309	4776	5309	5909	6407	6918	7442	7978	8530	8747	8964
Technical Officer (Laboratory)	637	719	791	883	956	1031	1109	1189	1272	1304	1337
TOTAL	***16398***	***18258***	***20281***	***22504***	***24451***	***26372***	***28350***	***30386***	***32484***	***33310***	***34137***

Supplementary Table 2: Aggregate HWF Forecast for Brong Ahafo Region

STAFF TYPE	2016	2017	2018	2019	2020	2021	2022	2023	2024	2025	2026
Biomedical Scientist	123	138	153	168	185	202	219	238	258	264	271
Community Health Nurse	1574	1732	1898	2081	2263	2394	2529	2669	2814	2886	2957
Critical Care Nurse	103	120	137	155	174	194	215	236	259	265	272
Dental Surgeon	25	27	28	30	32	34	36	38	40	41	42
Emergency Nurse	116	130	145	161	177	194	212	230	250	256	262
Enrolled Nurse	1841	2003	2164	2352	2532	2688	2849	3014	3184	3265	3346
Family Medicine Physician	24	25	27	28	29	31	33	34	36	37	38
General Surgeon	26	28	30	33	35	38	40	43	46	47	48
Medical Officer (General Practitioner)	355	385	411	454	490	527	566	607	648	665	681
Mental Health Nurse	225	240	249	276	293	312	332	352	373	382	392
Midwife	1009	1101	1182	1298	1399	1502	1608	1718	1830	1877	1924
Obstetrician & Gynaecologist	54	59	65	71	77	83	90	96	103	106	108
Ophthalmic Nurse	48	53	57	62	67	72	77	82	88	90	93
Ophthalmologist	11	12	14	15	17	18	20	21	23	24	24
Paediatrician	49	54	59	64	69	74	80	85	91	93	95
Pharmacist	94	104	115	125	136	148	160	173	186	191	196
Pharmacy Technician	418	452	479	525	564	603	644	687	731	749	768
Physician Assistant (Anaesthesia)	90	98	105	114	122	131	140	150	160	164	168
Physician Assistant (Medical)	276	296	310	342	366	392	418	445	474	486	498
Public Health Nurse	107	115	125	134	144	154	164	175	186	190	195
Radiographer /X-ray Technician	81	88	96	103	111	119	128	137	146	149	153
Registered General Nurse	2207	2425	2629	2891	3133	3384	3644	3914	4195	4302	4409
Technical Officer (Laboratory)	443	478	514	552	592	632	674	718	762	782	801
TOTAL	***9301***	***10166***	***10991***	***12035***	***13006***	***13926***	***14877***	***15862***	***16883***	***17313***	***17742***

Supplementary Table 3: Aggregate HWF Forecast for Central Region

STAFF TYPE	2016	2017	2018	2019	2020	2021	2022	2023	2024	2025	2026
Biomedical Scientist	152	166	187	212	226	241	256	273	290	298	305
Community Health Nurse	1822	2020	2262	2489	2699	2859	3027	3202	3385	3471	3557
Critical Care Nurse	158	171	188	206	221	236	252	270	289	296	304
Dental Surgeon	23	24	27	28	30	32	34	37	39	40	41
Emergency Nurse	121	133	149	168	181	196	212	229	247	254	260
Enrolled Nurse	1658	1849	2080	2303	2519	2714	2915	3124	3339	3424	3509
Family Medicine Physician	24	25	27	29	31	32	34	36	38	39	40
General Surgeon	29	30	33	35	37	39	42	44	47	48	50
Medical Officer (General Practitioner)	259	293	329	381	419	458	500	545	591	606	621
Mental Health Nurse	170	188	204	235	254	276	298	321	345	354	363
Midwife	795	901	1016	1163	1274	1389	1508	1632	1759	1804	1849
Obstetrician & Gynaecologist	48	53	59	65	71	78	84	91	99	101	104
Ophthalmic Nurse	41	48	57	68	73	78	83	89	94	97	99
Ophthalmologist	9	9	10	11	13	14	16	18	20	20	21
Paediatrician	44	48	54	59	65	70	76	82	89	91	93
Pharmacist	101	111	125	140	151	162	173	185	198	203	208
Pharmacy Technician	344	380	424	484	523	564	608	654	701	719	737
Physician Assistant (Anaesthesia)	72	79	88	96	105	115	125	136	147	151	154
Physician Assistant (Medical)	201	227	254	301	325	351	378	407	437	448	460
Public Health Nurse	77	88	103	120	131	142	154	166	179	184	188
Radiographer /X-ray Technician	70	79	91	104	113	121	130	139	149	153	156
Registered General Nurse	2157	2374	2641	2953	3194	3447	3711	3988	4277	4386	4495
Technical Officer (Laboratory)	344	394	468	554	595	639	684	731	779	799	819
TOTAL	***8718***	***9690***	***10877***	***12203***	***13246***	***14252***	***15302***	***16398***	***17541***	***17987***	***18434***

Supplementary Table 4: Aggregate HWF Forecast for Eastern Region

STAFF TYPE	2016	2017	2018	2019	2020	2021	2022	2023	2024	2025	2026
Biomedical Scientist	112	130	145	161	181	199	217	237	257	264	270
Community Health Nurse	1837	2018	2197	2399	2605	2758	2916	3078	3246	3328	3411
Critical Care Nurse	96	114	131	149	170	190	211	233	256	263	269
Dental Surgeon	29	31	32	33	36	38	39	41	43	44	45
Emergency Nurse	105	121	135	151	170	188	206	226	247	253	259
Enrolled Nurse	1967	2175	2358	2574	2803	2991	3182	3376	3573	3664	3755
Family Medicine Physician	27	30	31	32	34	36	37	39	40	41	42
General Surgeon	30	32	34	36	39	41	44	46	49	50	51
Medical Officer (General Practitioner)	348	385	410	459	503	546	590	637	685	702	720
Mental Health Nurse	235	256	265	296	320	343	367	391	416	427	437
Midwife	973	1094	1187	1325	1456	1582	1709	1838	1969	2019	2069
Obstetrician & Gynaecologist	53	59	65	72	79	86	93	101	108	111	114
Ophthalmic Nurse	44	50	54	59	66	71	76	82	88	90	93
Ophthalmologist	8	9	11	12	14	16	18	21	23	23	24
Paediatrician	48	55	60	66	73	79	85	91	97	100	102
Pharmacist	87	99	110	122	136	148	161	175	189	194	199
Pharmacy Technician	410	454	483	536	587	634	682	731	782	802	822
Physician Assistant (Anaesthesia)	89	99	107	115	127	136	147	157	168	172	177
Physician Assistant (Medical)	266	292	306	344	375	405	437	469	503	516	529
Public Health Nurse	98	110	120	131	144	156	168	180	192	197	202
Radiographer /X-ray Technician	77	86	94	101	112	121	130	139	149	153	156
Registered General Nurse	2152	2433	2655	2950	3261	3545	3836	4135	4444	4557	4670
Technical Officer (Laboratory)	391	439	479	520	575	621	668	717	767	787	806
TOTAL	***9480***	***10569***	***11469***	***12642***	***13868***	***14929***	***16019***	***17140***	***18293***	***18759***	***19224***

Supplementary Table 5: Aggregate HWF Forecast for Greater Accra Region

STAFF TYPE	2016	2017	2018	2019	2020	2021	2022	2023	2024	2025	2026
Biomedical Scientist	232	258	284	304	323	342	362	382	403	413	424
Community Health Nurse	1616	1846	2116	2318	2560	2757	2965	3184	3416	3503	3590
Critical Care Nurse	273	299	324	347	370	393	417	442	468	479	491
Dental Surgeon	21	23	25	27	28	30	32	34	36	36	37
Emergency Nurse	170	189	208	225	241	259	277	296	316	324	332
Enrolled Nurse	1449	1662	1902	2103	2325	2528	2739	2959	3187	3268	3349
Family Medicine Physician	29	31	34	35	37	39	41	42	44	46	47
General Surgeon	34	37	40	42	45	47	50	53	56	57	59
Medical Officer (General Practitioner)	248	285	320	359	395	434	475	518	564	578	592
Mental Health Nurse	112	131	147	168	187	206	227	249	273	280	287
Midwife	805	923	1039	1158	1270	1385	1504	1628	1755	1799	1844
Obstetrician & Gynaecologist	59	66	73	79	86	92	100	107	115	118	120
Ophthalmic Nurse	54	62	70	76	80	85	89	94	99	102	104
Ophthalmologist	12	13	15	17	18	20	22	24	27	27	28
Paediatrician	51	56	63	69	75	81	87	93	100	103	105
Pharmacist	152	168	185	199	212	225	239	253	268	274	281
Pharmacy Technician	273	318	359	400	437	476	517	561	606	621	637
Physician Assistant (Anaesthesia)	74	82	92	100	108	117	127	137	148	151	155
Physician Assistant (Medical)	121	147	168	192	212	233	256	281	308	316	324
Public Health Nurse	77	90	103	113	123	133	144	154	165	169	174
Radiographer /X-ray Technician	79	91	102	110	118	126	134	143	152	156	159
Registered General Nurse	2754	3069	3382	3677	3958	4249	4547	4855	5172	5304	5436
Technical Officer (Laboratory)	313	376	435	477	510	545	582	619	658	675	692
TOTAL	***9007***	***10222***	***11484***	***12597***	***13719***	***14803***	***15933***	***17110***	***18334***	***18801***	***19267***

Supplementary Table 6: Aggregate HWF Forecast for Northern Region

STAFF TYPE	2016	2017	2018	2019	2020	2021	2022	2023	2024	2025	2026
Biomedical Scientist	142	151	164	174	276	290	305	321	337	346	354
Community Health Nurse	1256	1441	1616	1816	2023	2168	2318	2475	2639	2706	2773
Critical Care Nurse	143	153	165	177	321	338	355	374	393	403	413
Dental Surgeon	19	20	22	24	25	27	28	30	32	33	33
Emergency Nurse	102	112	124	136	209	223	239	255	273	280	287
Enrolled Nurse	1341	1524	1709	1912	2101	2266	2436	2609	2787	2858	2929
Family Medicine Physician	22	23	25	26	32	33	35	36	37	38	39
General Surgeon	24	26	28	30	39	41	43	45	47	49	50
Medical Officer (General Practitioner)	264	295	320	361	434	470	507	545	585	600	615
Mental Health Nurse	186	206	217	244	272	290	310	330	351	360	369
Midwife	797	894	980	1095	1360	1464	1569	1676	1786	1831	1877
Obstetrician & Gynaecologist	42	46	52	57	77	83	89	95	102	104	107
Ophthalmic Nurse	38	42	47	51	65	69	74	78	83	85	87
Ophthalmologist	8	9	10	11	17	19	20	22	24	24	25
Paediatrician	40	44	49	54	69	74	79	85	90	92	95
Pharmacist	94	101	111	119	182	192	203	214	225	231	237
Pharmacy Technician	328	364	396	440	513	552	592	633	676	693	710
Physician Assistant (Anaesthesia)	60	67	76	83	98	107	115	124	134	137	140
Physician Assistant (Medical)	200	224	241	273	297	322	348	375	404	414	424
Public Health Nurse	69	77	88	97	109	118	128	138	149	153	156
Radiographer /X-ray Technician	63	68	76	83	102	109	116	124	132	136	139
Registered General Nurse	2082	2275	2477	2705	3829	4065	4307	4557	4815	4937	5060
Technical Officer (Laboratory)	304	334	377	411	447	484	523	563	604	619	635
TOTAL	***7623***	***8494***	***9369***	***10377***	***12897***	***13803***	***14739***	***15706***	***16705***	***17130***	***17555***

Supplementary Table 7: Aggregate HWF Forecast for Upper East Region

STAFF TYPE	2016	2017	2018	2019	2020	2021	2022	2023	2024	2025	2026
Biomedical Scientist	74	84	99	112	125	138	153	168	183	188	193
Community Health Nurse	1583	1725	1838	2030	2191	2297	2405	2515	2627	2694	2761
Critical Care Nurse	59	71	87	100	114	129	144	160	177	182	186
Dental Surgeon	15	16	18	19	20	21	22	24	25	26	27
Emergency Nurse	65	73	85	95	106	118	131	144	158	162	166
Enrolled Nurse	1451	1579	1699	1876	2024	2148	2274	2404	2538	2602	2667
Family Medicine Physician	15	16	18	19	20	21	23	24	25	26	27
General Surgeon	16	18	20	21	23	25	26	28	30	31	32
Medical Officer (General Practitioner)	316	338	350	391	419	448	478	510	543	557	571
Mental Health Nurse	251	269	275	310	330	351	372	394	416	426	437
Midwife	785	863	924	1042	1131	1224	1318	1416	1516	1554	1593
Obstetrician & Gynaecologist	29	32	37	41	45	49	54	58	63	65	67
Ophthalmic Nurse	31	34	38	41	45	49	53	58	62	64	66
Ophthalmologist	4	5	6	7	8	9	11	12	13	14	14
Paediatrician	25	28	33	36	40	44	48	52	57	58	59
Pharmacist	55	62	72	80	89	98	108	118	128	131	135
Pharmacy Technician	359	393	419	471	510	550	592	634	678	696	713
Physician Assistant (Anaesthesia)	48	53	59	65	70	76	82	89	96	99	101
Physician Assistant (Medical)	289	313	325	367	395	424	453	484	516	529	542
Public Health Nurse	58	64	72	79	86	93	101	109	118	121	124
Radiographer /X-ray Technician	47	52	59	64	70	76	82	89	97	99	102
Registered General Nurse	1604	1787	1971	2220	2429	2646	2869	3099	3337	3422	3507
Technical Officer (Laboratory)	260	286	326	358	392	428	466	506	549	563	577
TOTAL	***7442***	***8162***	***8830***	***9846***	***10682***	***11461***	***12265***	***13095***	***13954***	***14309***	***14664***

Supplementary Table 8: Aggregate HWF Forecast for Upper West Region

STAFF TYPE	2016	2017	2018	2019	2020	2021	2022	2023	2024	2025	2026
Biomedical Scientist	47	55	63	71	79	88	97	106	116	119	122
Community Health Nurse	941	1066	1166	1299	1424	1495	1567	1641	1717	1761	1804
Critical Care Nurse	48	58	68	78	89	100	111	123	135	138	142
Dental Surgeon	11	11	12	12	12	13	13	14	14	15	15
Emergency Nurse	56	62	68	75	81	88	96	103	111	114	117
Enrolled Nurse	948	1037	1102	1201	1287	1347	1409	1472	1536	1576	1615
Family Medicine Physician	10	10	11	11	12	12	13	14	14	15	15
General Surgeon	12	13	14	15	15	16	17	18	20	20	21
Medical Officer (General Practitioner)	202	219	224	246	261	276	292	309	326	334	342
Mental Health Nurse	121	135	137	156	168	180	192	205	218	224	229
Midwife	477	526	551	611	656	703	751	800	851	872	894
Obstetrician & Gynaecologist	25	27	29	31	33	35	38	40	42	43	44
Ophthalmic Nurse	19	20	21	23	25	26	28	30	32	33	34
Ophthalmologist	5	6	7	7	8	9	9	10	11	11	11
Paediatrician	21	23	24	26	28	29	31	33	35	36	37
Pharmacist	40	44	49	54	60	65	71	76	83	85	87
Pharmacy Technician	183	205	215	243	263	284	306	328	352	361	369
Physician Assistant (Anaesthesia)	42	44	46	48	51	53	56	58	61	63	64
Physician Assistant (Medical)	132	149	154	176	191	207	224	241	259	265	272
Public Health Nurse	43	45	47	49	52	54	57	60	63	65	66
Radiographer /X-ray Technician	32	34	36	38	41	44	46	49	52	54	55
Registered General Nurse	1003	1127	1216	1360	1482	1608	1739	1873	2011	2062	2114
Technical Officer (Laboratory)	144	154	165	176	189	202	217	232	249	255	261
TOTAL	***4560***	***5069***	***5426***	***6008***	***6506***	***6936***	***7379***	***7836***	***8308***	***8519***	***8731***

Supplementary Table 9: Aggregate HWF Forecast for Volta Region

STAFF TYPE	2016	2017	2018	2019	2020	2021	2022	2023	2024	2025	2026
Biomedical Scientist	98	110	123	137	152	167	183	200	218	223	229
Community Health Nurse	1571	1748	1894	2119	2317	2464	2616	2772	2934	3008	3083
Critical Care Nurse	78	92	107	123	139	156	174	193	213	218	224
Dental Surgeon	25	26	27	28	29	31	32	34	36	36	37
Emergency Nurse	85	95	107	120	134	149	165	182	199	204	209
Enrolled Nurse	1652	1839	1993	2220	2417	2591	2767	2947	3130	3210	3290
Family Medicine Physician	25	26	27	28	29	31	32	33	34	35	36
General Surgeon	26	28	29	31	32	34	36	38	41	42	43
Medical Officer (General Practitioner)	337	368	384	433	468	506	545	586	628	644	660
Mental Health Nurse	246	267	272	309	331	354	378	402	427	438	449
Midwife	852	965	1046	1191	1306	1423	1541	1662	1784	1829	1875
Obstetrician & Gynaecologist	41	46	51	57	63	69	75	81	88	90	92
Ophthalmic Nurse	37	42	46	50	55	59	64	69	74	76	78
Ophthalmologist	5	6	8	9	11	12	14	16	18	18	19
Paediatrician	37	42	47	52	58	63	68	74	79	81	84
Pharmacist	74	82	92	102	112	123	134	146	159	163	167
Pharmacy Technician	378	418	443	501	545	590	638	686	736	755	774
Physician Assistant (Anaesthesia)	71	78	85	92	100	108	117	126	135	139	142
Physician Assistant (Medical)	262	289	301	345	374	405	438	471	506	519	531
Public Health Nurse	78	87	96	105	115	125	136	146	157	161	165
Radiographer /X-ray Technician	65	71	77	84	91	99	106	115	123	126	130
Registered General Nurse	1882	2124	2320	2615	2870	3130	3397	3670	3951	4052	4152
Technical Officer (Laboratory)	324	357	393	431	470	512	555	601	648	665	681
TOTAL	***8250***	***9207***	***9969***	***11182***	***12219***	***13202***	***14212***	***15250***	***16319***	***16734***	***17149***

Supplementary Table 10: Aggregate HWF Forecast for Western Region

STAFF TYPE	2016	2017	2018	2019	2020	2021	2022	2023	2024	2025	2026
Biomedical Scientist	98	115	129	147	163	179	195	213	231	237	242
Community Health Nurse	2153	2338	2553	2730	2938	3095	3256	3422	3594	3685	3777
Critical Care Nurse	81	99	115	134	152	170	189	209	230	236	242
Dental Surgeon	25	27	28	31	32	33	35	37	39	40	41
Emergency Nurse	89	104	117	134	149	166	183	201	220	226	231
Enrolled Nurse	1979	2182	2379	2593	2801	2984	3170	3359	3551	3641	3732
Family Medicine Physician	25	27	28	30	31	33	34	35	36	37	38
General Surgeon	26	29	30	33	35	37	39	41	44	45	46
Medical Officer (General Practitioner)	270	306	334	382	422	464	508	553	600	616	631
Mental Health Nurse	179	199	211	240	262	285	308	332	358	367	376
Midwife	799	913	1006	1143	1259	1377	1497	1619	1743	1788	1832
Obstetrician & Gynaecologist	46	52	58	65	71	77	84	91	98	100	102
Ophthalmic Nurse	38	44	48	54	58	63	68	73	78	80	82
Ophthalmologist	7	8	9	11	13	15	17	19	21	21	22
Paediatrician	43	49	54	60	66	71	77	82	88	90	93
Pharmacist	77	89	99	112	123	134	146	158	171	175	179
Pharmacy Technician	332	373	402	456	497	541	586	632	681	698	716
Physician Assistant (Anaesthesia)	78	87	94	105	113	122	131	141	151	155	159
Physician Assistant (Medical)	204	228	244	278	305	333	363	394	427	438	449
Public Health Nurse	86	97	106	118	129	139	150	161	172	177	181
Radiographer /X-ray Technician	67	76	82	92	100	108	116	124	133	136	140
Registered General Nurse	1822	2086	2302	2608	2865	3128	3397	3674	3959	4060	4160
Technical Officer (Laboratory)	342	386	421	470	509	550	591	634	679	696	713
TOTAL	***8865***	***9911***	***10850***	***12027***	***13092***	***14103***	***15141***	***16207***	***17304***	***17744***	***18185***
